# PPP2R1A silencing suppresses LUAD progression by sensitizing cells to nelfinavir-induced apoptosis and pyroptosis

**DOI:** 10.1186/s12935-024-03321-5

**Published:** 2024-04-23

**Authors:** Yating Liu, Lianlian Ouyang, Shiyao Jiang, Lu Liang, Yuanbing Chen, Chao Mao, Yiqun Jiang, Li Cong

**Affiliations:** 1 Department of Pharmacy, The Third Xiangya Hospital, Central South University, Changsha, 410013 Hunan China; 2 Department of Dermatology, Hunan Key Laboratory of Medical Epigenomics, Second Xiangya Hospital, Central South University, Changsha, 410011 China; 3 Research Unit of Key Technologies of Diagnosis and Treatment for Immune-Related Skin Diseases, Chinese Academy of Medical Sciences, Changsha, 410011 China; 4 The Key Laboratory of Model Animal and Stem Cell Biology in Hunan Province, Hunan Normal University, Changsha, 410013 Hunan People’s Republic of China; 5 School of Medicine, Hunan Normal University, Changsha, 410013 Hunan People’s Republic of China; 6 Department of Neurosurgery, The Third Xiangya Hospital, Central South University, Changsha, 410013 Hunan China; 7 Department of Experimental Radiation Oncology, The University of Texas MD Anderson Cancer Center, Houston, TX USA

**Keywords:** Lung adenocarcinoma, Meiotic genes, Prognostic model, PPP2R1A, Nelfinavir

## Abstract

Lung adenocarcinoma is a major public health problem with the low 5-year survival rate (15%) among cancers. Aberrant alterations of meiotic genes, which have gained increased attention recently, might contribute to elevated tumor risks. However, systematic and comprehensive studies based on the relationship between meiotic genes and LUAD recurrence and treatment response are still lacking. In this manuscript, we first confirmed that the meiosis related prognostic model (MRPM) was strongly related to LUAD progression via LASSO-Cox regression analyses. Furthermore, we identified the role of PPP2R1A in LUAD, which showed more contributions to LUAD process compared with other meiotic genes in our prognostic model. Additionally, repression of PPP2R1A enhances cellular susceptibility to nelfinavir-induced apoptosis and pyroptosis. Collectively, our findings indicated that meiosis-related genes might be therapeutic targets in LUAD and provided crucial guidelines for LUAD clinical intervention.

**Graphical Abstract:**

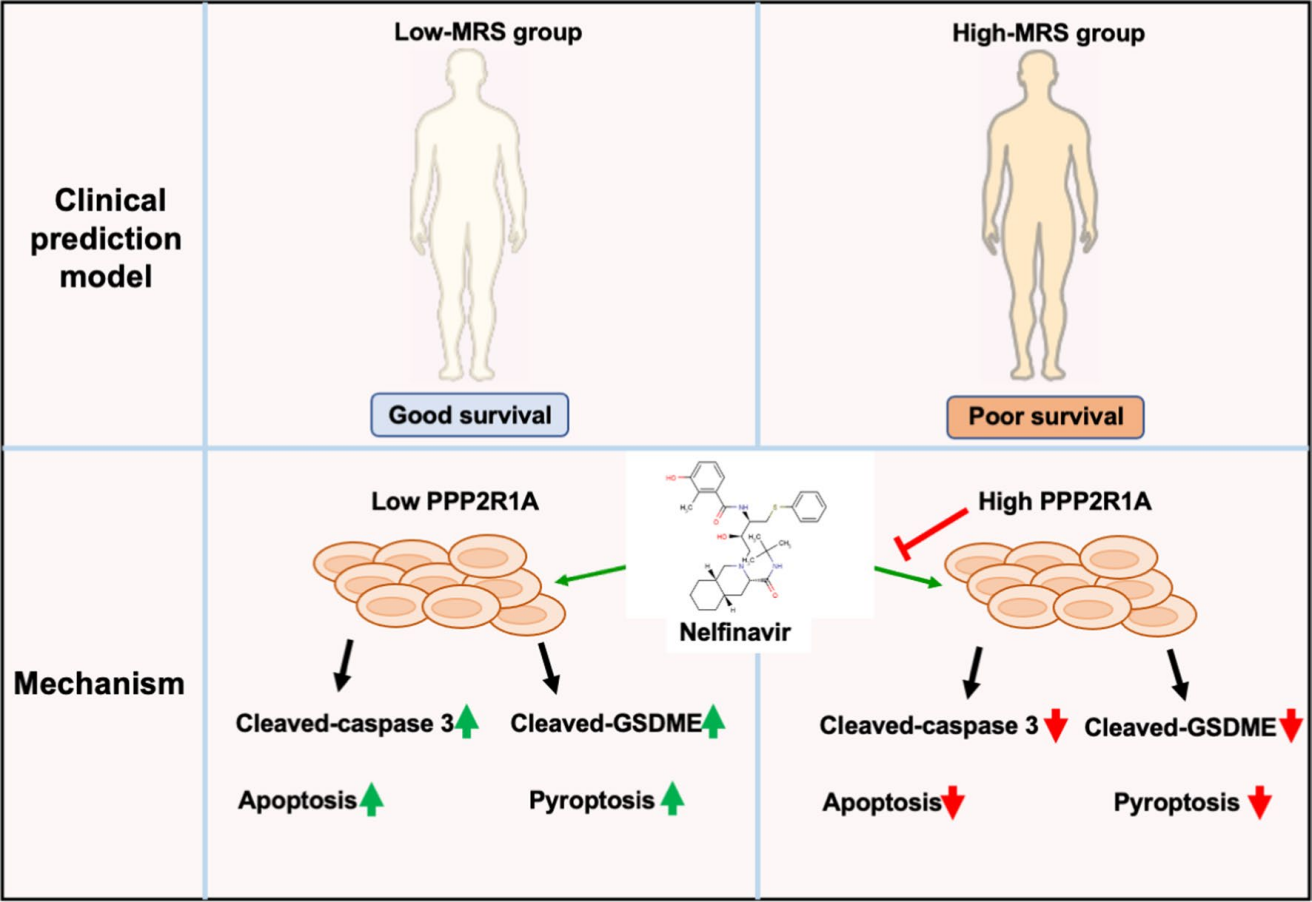

**Supplementary Information:**

The online version contains supplementary material available at 10.1186/s12935-024-03321-5.

## Highlights

- Meiotic genes-related risk score (MRS) is a promising prediction feature with high reliability and accuracy for LUAD patients.
- PPP2R1A is overexpressed in LUAD and associated with malignancy.
- Nelfinavir inhibited cell viability and yields anti-tumor effects in LUAD cells.
- PPP2R1A silencing sensitizes cancer cells to nelfinavir induced cell apoptosis and pyroptosis.

**Supplementary Information:**

The online version contains supplementary material available at 10.1186/s12935-024-03321-5.

## Introduction

Lung cancer is the leading cause of cancer-related death worldwide [1, 2]. Lung adenocarcinoma (LUAD) is the most common histological type of lung cancer, which accounts for 40% of lung cancer. Due to its late diagnosis, tumor high heterogeneity and therapy resistance, LUAD remains a major public health problem with the low 5-year survival rate (15%) among cancers [3]. At present, large-scale genomic studies of human tumor biopsies have defined several genetic alterations in the initiation and progress of LUAD such as TP53, KRAS and EGFR [3, 4]. Combined with drug development and the molecular characterization, LUAD patients benefit from precision therapies that targeted EGFR or KRAS. However, the heterogeneity of the LUAD limits the therapy treatment and prognosis of each patient precisely. Therefore, how to guide the selection of effective and sensitive treatment of LUAD patients remains a great challenge.

Double-strand break (DSB), a serious type of DNA damage generated by physiological or pathological means, triggers multiple responses within cells [5, 6]. Once DSB occurs near or at the transcription site, transcriptional repression was induced by DSB [7]. In recent years, several signaling pathways and transcriptional factors were reported to control DSB-induced transcriptional repression, including ATM signaling [8] and DNA-PKcs signaling [9]. Cancer cells possess abnormal DSB repair to promote cell proliferation, provide survival microenvironment and increase resistance. It is well known that various cancers possess alteration of DSB repair genes. Activation of DSB repair genes is a challenge for chemoresistance and radio-resistance.

Recent studies have found that aberrant regulation of meiotic genes in somatic cancers affect homologous recombination (HR)-dependent-DNA repair [10]. HORMAD1 is one of the most studied meiotic genes implicated in carcinogenesis and genomic instability [11]. HORMAD1 is significantly upregulated in several cancers, including breast cancer, lung cancer [12], colon cancer, gastric cancer and melanoma [13, 14], which contributes to increasing genomic instability and poor patient prognosis. PRDM9 is also a gene that involved in meiotic recombination, and was found to be expressed in various tumors. Intriguingly, the expression of PRDM9 was closely correlated with genomic instability [15, 16]. DMC1, another meiosis-specific gene, has been reported to drive cancer cells escape from cell death induced by radiation and drug. DMC1 inhibition also contributes to reduced tumor growth and prolonged survival in vivo [17–19]. All these investigations have suggested that the meiotic repair genes provide tumor cells with a repair mechanism to increase resistance and evade cell death caused by DNA damage. These observations also raised our interest for targeting meiotic genes that are aberrantly expressed in cancer cells.

In the present study, we identified meiosis-related genes in LUAD and constructed a meiosis related prognostic model by Cox regression and least absolute shrinkage and selection operator (LASSO) regression analyses based on TCGA database, which showed high accuracy in predicting recurrence and was confirmed by GSE31210. Furthermore, we identified the role of PPP2R1A in LUAD, which showed more contributions to LUAD process compared with other meiotic genes in our prognostic model. PPP2R1A was shown to promote cell proliferation and drug-resistance in LUAD cell lines. Moreover, the interaction between the antiretroviral drug nelfinavir and PPP2R1A protein was predicted. Repression of PPP2R1A enhances cellular susceptibility to nelfinavir treatment. Additionally, nelfinavir combined with cisplatin exhibited enhanced efficacy in inducing cell apoptosis and pyroptosis.

Collectively, these findings indicated that meiosis-related genes might be therapeutic targets in LUAD and provided crucial guidelines for LUAD clinical intervention.

## Results

### Identification and analysis of meiotic genes in lung adenocarcinoma

**Fig. 1:**
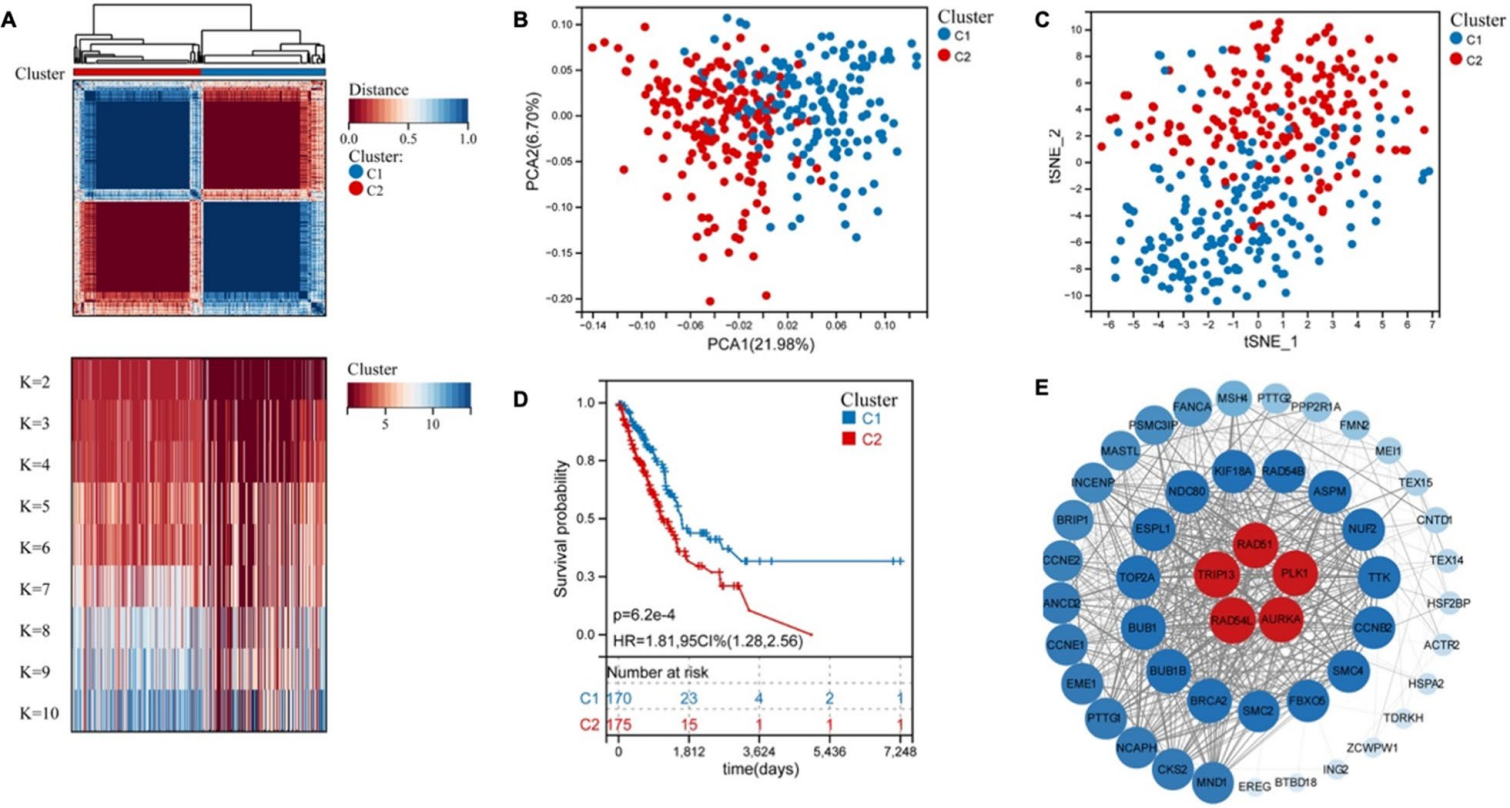
50 meiotic genes associated with prognosis of lung adenocarcinoma were identified. **A** K-means clustering analysis of meiosis related genes was performed using the expression profile of lung adenocarcinoma in TCGA; heatmap displaying consensus clustering with the robust classification (k = 2). **B** PCA plot proved the feasibility of grouping. **C** tSNE plot proved the feasibility of grouping. **D** Kaplan-Meier curves of OS between clusters. Log-rank test p values are shown. **E** A PPI network was constructed based on 50 prognostic-related genes obtained by univariate regression analysis

To systematically and comprehensively examine the predict prognosis role of meiotic genes in LUAD, we present the application of K-means clustering method for the classification of LUAD samples from TCGA database based on the expression of meiosis related genes. As shown in Fig. 1A, the following two distinct patterns were the most valid: 170 cases in Cluster1 (C1) and 175 cases in Cluster2 (C2). Next, both the principal component analysis (PCA) plot and t-distributed stochastic neighbor embedding (tSNE) plot have proved the feasibility of grouping (Fig. 1B, C). The Kaplan–Meier (KM) survival analysis of the overall survival (OS) with the two subtypes showed significant differences (Fig. 1D). A protein–protein interaction (PPI) network was also constructed based on 50 prognostic-related genes obtained by univariate regression analysis (Fig. 1E). These 50 meiotic genes were considered to play more important roles in the progression of LUAD. Collectively, these results demonstrated that meiotic genes play important roles in LUAD recurrence and progression.

### Construction of a meiosis related prognostic model to predict the overall survival of LUAD patients

**Fig. 2:**
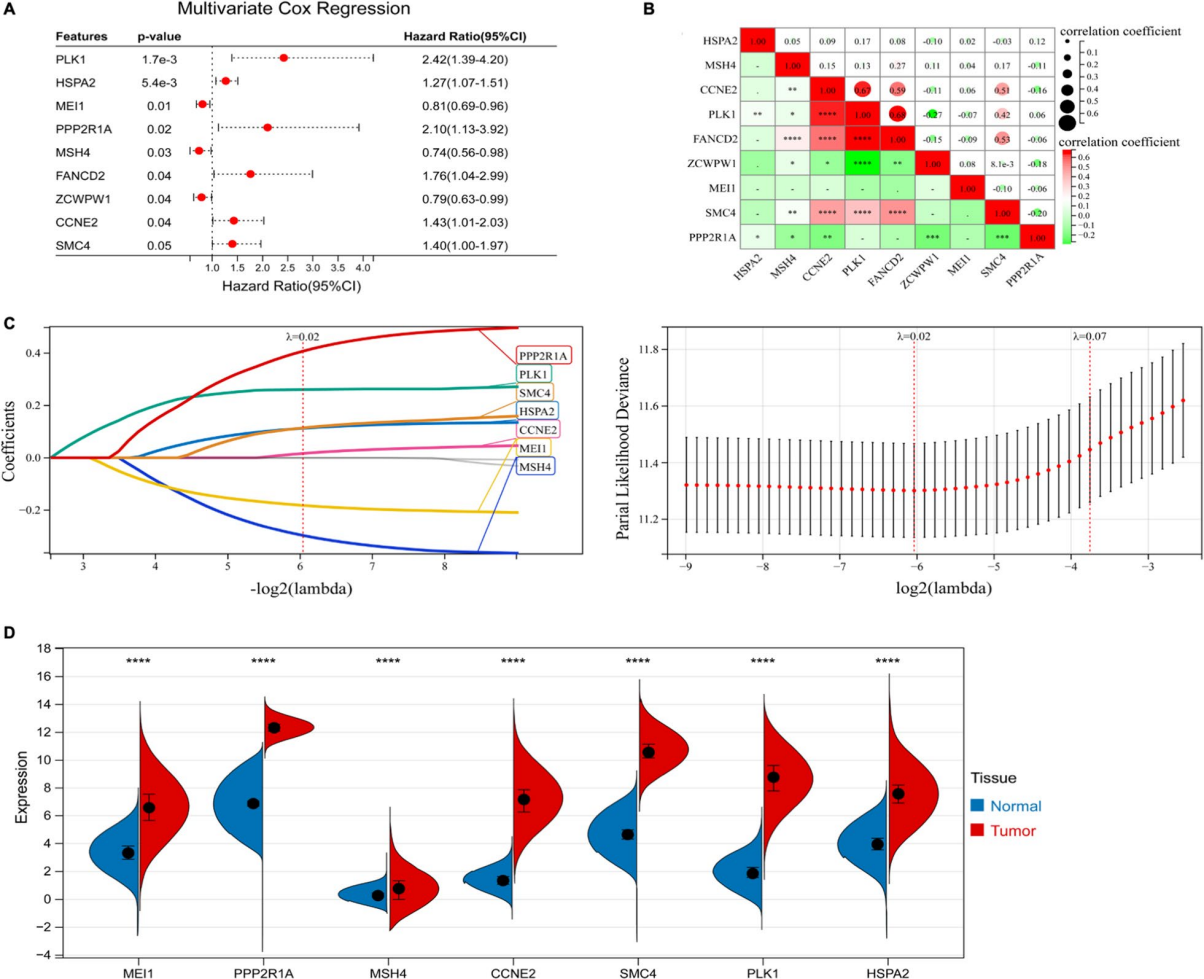
A meiosis related prognostic model was established in the TCGA-LUAD cohort. **A** Forest plot showed nine genes associated with OS using multivariate cox regression analysis. **B** Correlation heatmap showed correlations between nine genes. **C** LASSO coefficient profiles of 7 DEGs Cross-validation revealed an optimum parameter in the LASSO model. **D** Violin plot showed the expression of seven genes in lung adenocarcinoma and normal tissues using TCGA-LUAD and GTEX. **** *p* < 0.0001, *** *p* < 0.001, ** *p* < 0.01, * *p* < 0.05

To screen the OS-related meiotic genes based on the 50 genes, the multivariate cox regression analysis was used. As shown in Fig. 2A, 9 meiotic genes were markedly related to OS based on p-value less than 0.05, including 6 genes (PLK1, HSPA2, PPP2R1A, FANCD2, CCNE2, SMC4) that showed hazardous factors with hazard ratios (95% CI) greater than 1, and 3 genes (MEI1, MSH4, ZCWPW1) showed protective roles with hazard ratios (95% CI) less than 1(Fig. 2A). Correlation heatmap showed that these genes were correlated with each other, suggesting that meiotic genes in LUAD are overall changes (Fig. 2B).

To establish a comprehensive and effective meiosis related prognostic model (MRPM), we performed LASSO Cox regression analysis for the OS-related key meiotic genes. After cross-validation, 7 key meiotic genes (PPP2R1A, PLK1, SMC4, HSPA2, CCNE2, MEI1 and MSH4) were identified (Fig. 2C). The MRPM for prognosis was constructed based on the following formula: meiotic genes-related risk score (MRS) = 0.4088 * the expression of PPP2R1A + 0.2605 * the expression of PLK1 + 0.1143 * the expression of SMC4 + 0.1128 * the expression of HSPA2 + 0.0169 * the expression of CCNE2− 0.1829 * the expression of MEI1 − 0.2980 * the expression of MSH4. We then used TCGA-LUAD and GTEX to investigate the expression of 7 key meiotic genes in LUAD tissues and normal tissues (Fig. 2D). PPP2R1A, MEI1, CCNE2, SMC4, PLK1 and HSPA2 were significantly upregulated in LUAD tissues compared to normal tissues.

**Fig. 3:**
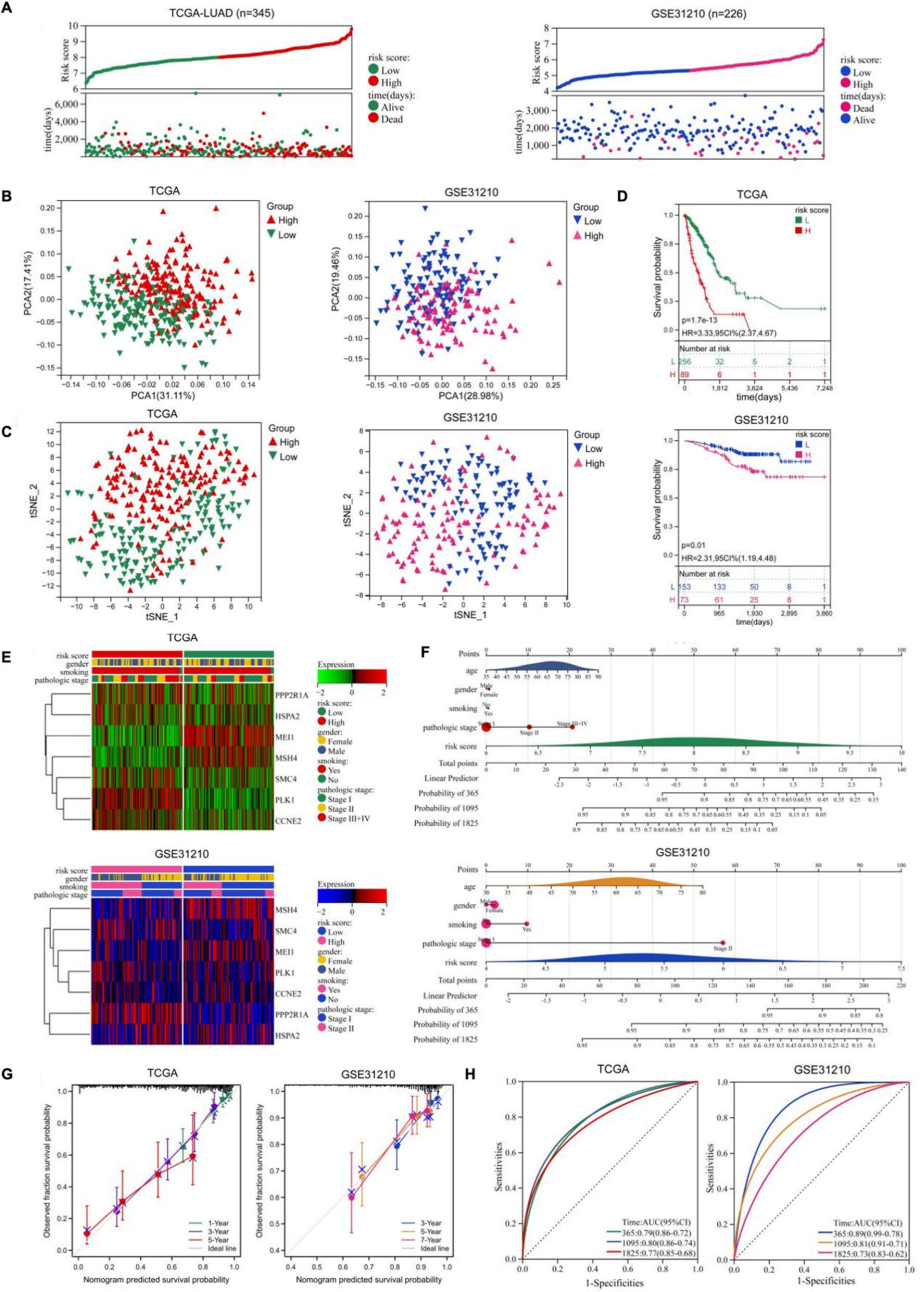
Evaluation of the meiosis related prognosis model in TCGA-LUAD and GSE31210. **A** Survival status and risk scores in TCGA and GSE31210. **B** PCA analysis of grouped samples in TCGA and GSE31210. **C** tSNE analysis of grouped samples in TCGA and GSE31210. **D** The KM curve showed longer OS in the low-risk group whether it was TCGA or GSE31210. **E** Unsupervised clustering of the 7 candidate genes using the tumor stage, smoking history, gender and risk score as patient annotations in TCGA and GSE31210. **F** Nomogram based on the 7 meiosis related genes quantitatively predicted the survival of patients with LUAD in TCGA and GSE31210. **G** Calibration plot for internal validation of the nomogram in TCGA and GSE31210. **H** AUC values of time-dependent ROC curves verified the predictive accuracy of the risk score in TCGA and GSE31210

We then calculated the MRS according to this model and divided the LUAD patients into a high-MRS subgroup and a low-MRS subgroup. As shown in Fig. 3A, the OS rate of LUAD patients with low-risk subgroup was obviously higher than that of the high-risk subgroup, indicating that higher MRS indicated a higher probability of recurrence. In addition, we also used GSE31210 datasets that contains 226 LUAD samples to test this prediction model. Similarly, the OS rate of low-risk subgroup in GSE31210 datasets was obviously higher than that of the high-risk subgroup (Fig. 3A). PCA, tSNE and UMAP analysis plot were demonstrated the validation of subgroup both in TCGA and GSE31210 (Fig. 3B, C, Additional file 1: Fig. S1A, B). Patients with high-risk score showed worse survival compared with patients with low-risk score both in TCGA and GSE31210 database (Fig. 3D). Besides, we explored the relationship among the risk score, clinical features (gender, smoking history and pathologic stage) and the expression levels of the seven key meiotic genes in TCGA and GSE31210 database. The heatmap plot of Fig. 3E showed that the expression of PPP2R1A, PLK1and SMC4 were upregulated in the high-risk subgroup both in TCGA and GSE31210 database, while the expression of MEI1 and MSH4 were downregulated in the high-risk subgroup. Next, we constructed a prediction model and visualized the model by nomogram (Fig. 3F). Both calibration plot and ROC curve analysis showed that the monogram model have great value for OS in TCGA and GSE31210 (Fig. 3G, H).

In summary, these findings demonstrated that the MRS may be a promising prediction feature with high reliability and accuracy for LUAD patients.

### Evaluating the characteristics of different meiotic genes-related risk score (MRS) subgroups

**Fig. 4:**
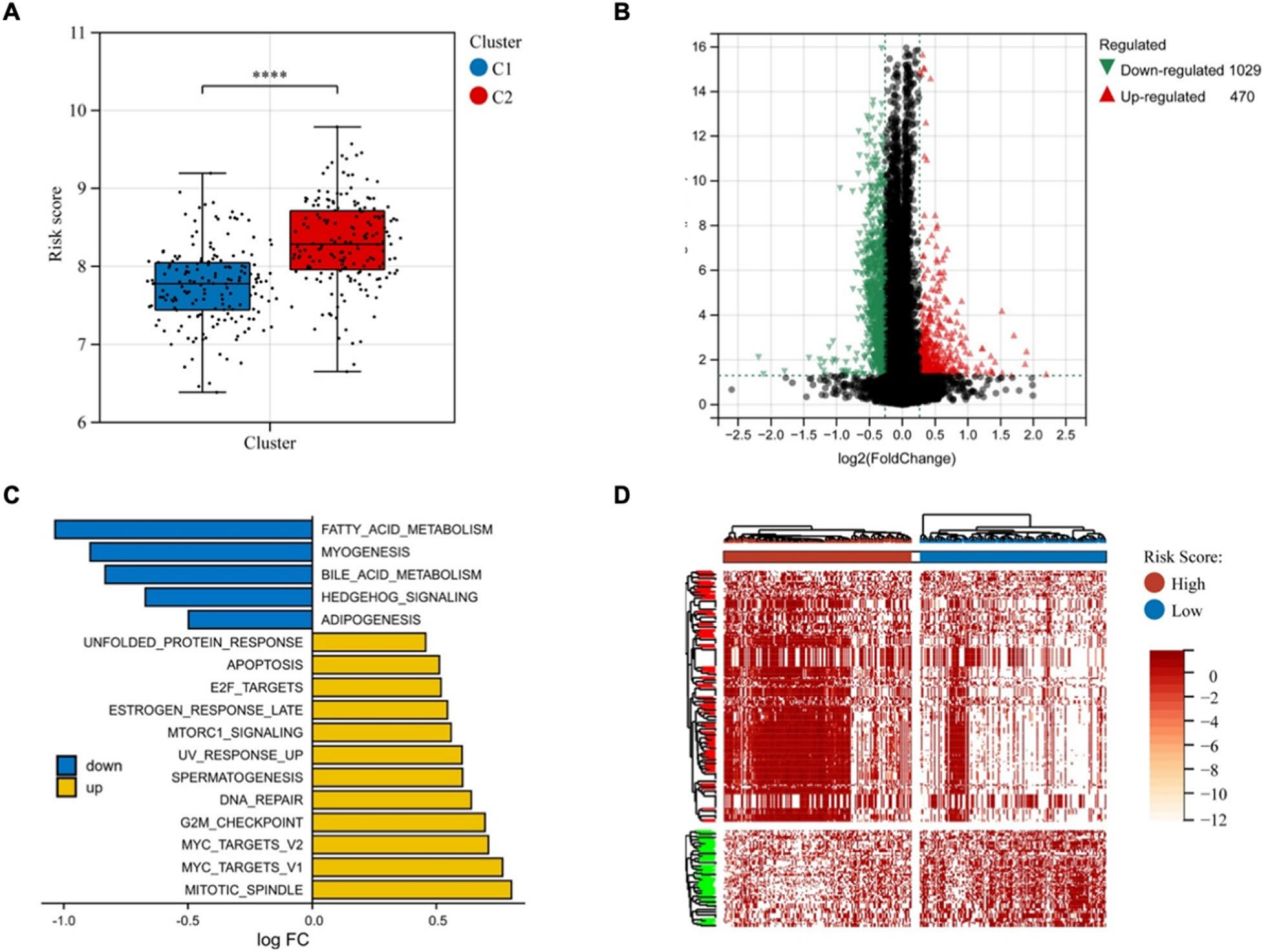
Identification of biological pathways associated with the risk of meiosis related prognosis model. **A** Boxplot showed the relationship between consistent clustering and risk score. **B** Volcano plot depicted DEGs in the high- and low-risk groups in the TCGA cohort (including 470 upregulated and 1029 downregulated genes). **C** Histogram showed pathway differences between high and low risk groups using hallmark gene sets by GSVA. **D** Heatmap showed pathway differences between high and low risk groups using C2 reactome gene sets by GSVA. *GSVA*: Gene Set Variation Analysis

To investigate the relationship between Cluster and risk score, box plot showed that Cluster 2 has a high-risk score compared with Cluster 1 (Fig. 4A). To systematically and comprehensively examine the difference at the gene level in LUAD TCGA database between the high-risk subgroup and the low-risk subgroup, we screened for differentially expressed genes (DEGs). Comparing the high-risk group to the low-risk group, 470 DEGs were upregulated and 1029 DEGs were downregulated in the TCGA cohort on the basis of p < 0.05 and |Fold change| > 1.2 (Fig. 4B). Gene set variation analysis (GSVA) was carried out to predict the gene set changes between the high- and low-risk groups in the LUAD TCGA cohort. The results revealed that the gene sets of the high-risk samples were gathered in pathways related to mitotic spindle, myc targets, G2M checkpoint, DNA repaire, UV response and mtorc1 signaling, while the low-risk group were enriched in fatty acid metabolism, myogenesis and bile acid metabolism, suggesting that there was a significant difference in tumor growth, metabolism, metastasis and therapy response (Fig. 4C). Moreover, the heatmap also showed a significant difference between the high and low risk groups using C2 reactome gene sets by GSVA (Fig. 4D). These results indicated that the subgroups had different microenvironments and response.

### Nelfinavir inhibited cell viability and yields anti-tumor effects in LUAD cells

**Fig. 5:**
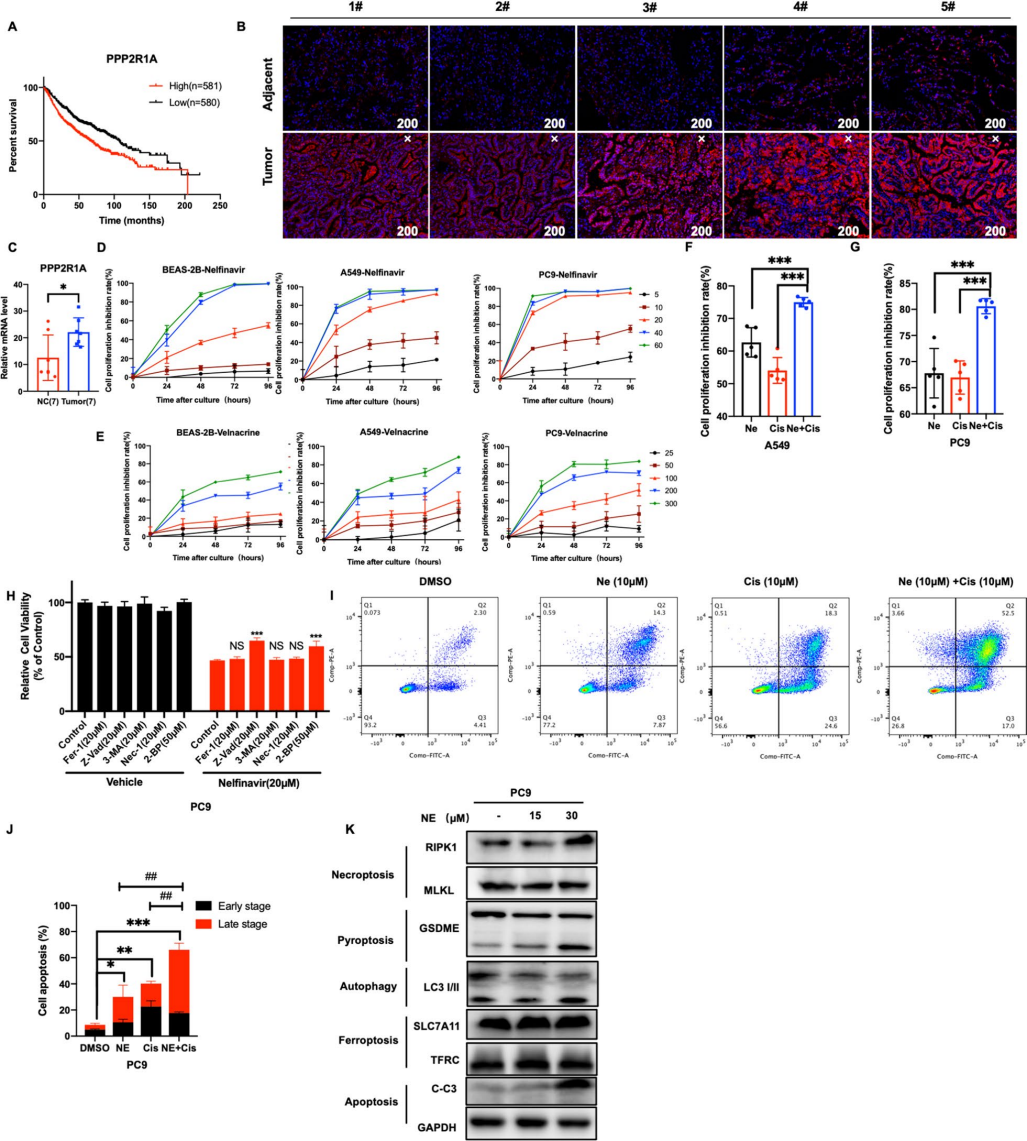
Nelfinavir inhibited cell viability and yields anti-tumor effects in LUAD cells. **A** Kaplan–Meier plotter showed the survival of LUAD patients in high expression group and low expression group of PPP2R1A. **B** mIHC showed the level of PPP2R1A in adjacent tissues and tumor tissues of LUAD patients (n = 5). **C** RT-qPCR was used to detect the mRNA level of PPP2R1A. **D** CCK8 assay measured the viability of BEAS-2B, A549 and PC9 cell lines under 0 (Control, DMSO), 5, 10, 20, 40 and 60 μmol nelfinavir treatment. **E** CCK8 assay measured the viability of BEAS-2B, A549 and PC9 cell lines under 0 (Control, DMSO), 25, 50, 100, 200, and 300 μmol velnacrine treatment. **F**,**G** CCK8 assay measured the viability of A549 (C) and PC9 (D) cell lines after 24 h of treatment with nelfinavir (10 μmol)/ cisplatin (10 μmol) or their combination. **H** Cell viability of PC9 cells treated with 20 μM Nelfinavir for 24 h in combination with ferroptosis inhibitor ferrostatin-1(Fer-1), apoptosis inhibitor Z-VAD-FMK, autophagy inhibitor 3-methyladenine(3-MA), necroptosis inhibitor necrostatin-1(Nec-1) and pyroptosis inhibitor 2-Bromohexadecanoic acid (2-BP). **I** Representative images of cell death after 24 h-treatment with nelfinavir (10 μmol)/ cisplatin (10 μmol) or their combination detected by flow cytometry. **J** Percentages of early-stage and late-stage apoptotic cells were compared among the four groups. **K** Western blot demonstrated that nelfinavir treatment induced the cleavage of caspase 3 and GSDME in PC9

Considering PPP2R1A revealed the greatest contribution to the MRS model, we next explored the expression and role of PPP2R1A in LUAD. Kaplan–Meier Plotter showed that high mRNA expression of PPP2R1A was closely correlated with poor survival of LUAD patients (Fig. 5A). We employed multiplex immunohistochemical (mIHC) and RT-qPCR to explore the protein and mRNA expression of PPP2R1A among tumor and adjacent tissues of LUAD patients (Fig. 5B, C). We found that PPP2R1A was highly expressed in adenocarcinoma tissues compared with adjacent tissues (Fig. 5B, C). These results were consistent with the data obtained from the TCGA database (Fig. 2D). Consistently, these results revealed that PPP2R1A is overexpressed in LUAD and associated with malignancy.

Using CMap, we identified the two chemicals—nelfinavir and velnacrine that might inhibit the effects of PPP2R1A and were correlated to reverse the risk score of LUAD patients (Additional file 1: Fig. S2A). To further investigate the effects of nelfinavir and velnacrine in LUAD, we treated BEAS-2B, A549 and PC9 cells with a series of concentration of nelfinavir and velnacrine in different timepoints. As shown in Fig. 5D, E, nelfinavir and velnacrine inhibited the viability of LUAD cells in a dose- and time- dependent manner. Even at a concentration as low as 10 μmol, nelfinavir significantly inhibited the proliferation of both A549 and PC9 cells. Velnacrine inhibited BEAS-2B, A549 and PC9 cell proliferation in a higher dose even at 100 μmol which limits the clinical use of velnacrine (Fig. 5E). BEAS-2B exhibits more tolerance to nelfinavir and velnacrine treatment compared with A549 and PC9 (Fig. 5D, E). Hence, we use nelfinavir for further investigation. CCK8 assay indicated that the combination of nelfinavir and cisplatin, a clinical chemotherapy drug for lung cancer therapy, suppressed cell proliferation more significantly than nelfinavir or cisplatin alone in cancer cells (Fig. 5F, G). These results demonstrated that a combination treatment of nelfinavir and cisplatin has a significant anti-tumor effect on cancer cells.

To investigate the type of cell death that occurred, multiple inhibitors of common cell death pathways, including apoptosis, pyroptosis, necroptosis, ferroptosis and autophagy, were used to rescue the cell death induced by nelfinavir treatment. Notably, the apoptosis inhibitor Z-VAD-FMK and pyroptosis inhibitor 2-Bromohexadecanoic acid (2-BP) reversed the reduced cell viability induced by nelfinavir treatment, but this finding was not obtained with the necroptosis inhibitor necrostatin-1, the autophagy inhibitor 3-methyladenine or the ferroptosis inhibitor ferrostatin-1 (Fig. 5H). To determine whether the cell proliferation inhibition induced by nelfinavir was associated with cell apoptosis, PC9 cells were treated with nelfinavir (10 μmol) and cisplatin (10 μmol) alone or in combination for 24 h and were subsequently analyzed via flow cytometry with Annexin V-FITC/PI. Significantly, nelfinavir combination with cisplatin increased cell death compared with nelfinavir/cisplatin alone (Fig. 5I–J). Combination treatment caused obvious cell death in late-stage, which could be associated with other cell death pathways (Fig. 5J). Next, we detected apoptosis, ferroptosis, autophagy, pyroptosis and necroptosis related protein through western blot. Interestingly, we found markedly increased cleaved forms of Caspase-3 and GSDME in PC9 cells treated with nelfinavir (Fig. 5K), but no obvious effect on necroptosis related protein RIPK1 and MLKL, autophagy related protein LC3-I/II or ferroptosis protein SLC7A11 and TFRC. Collectively, these data indicated that apoptosis and pyroptosis were induced by nelfinavir in LUAD cells.

### PPP2R1A regulates the drug resistance of LUAD cells

**Fig. 6:**
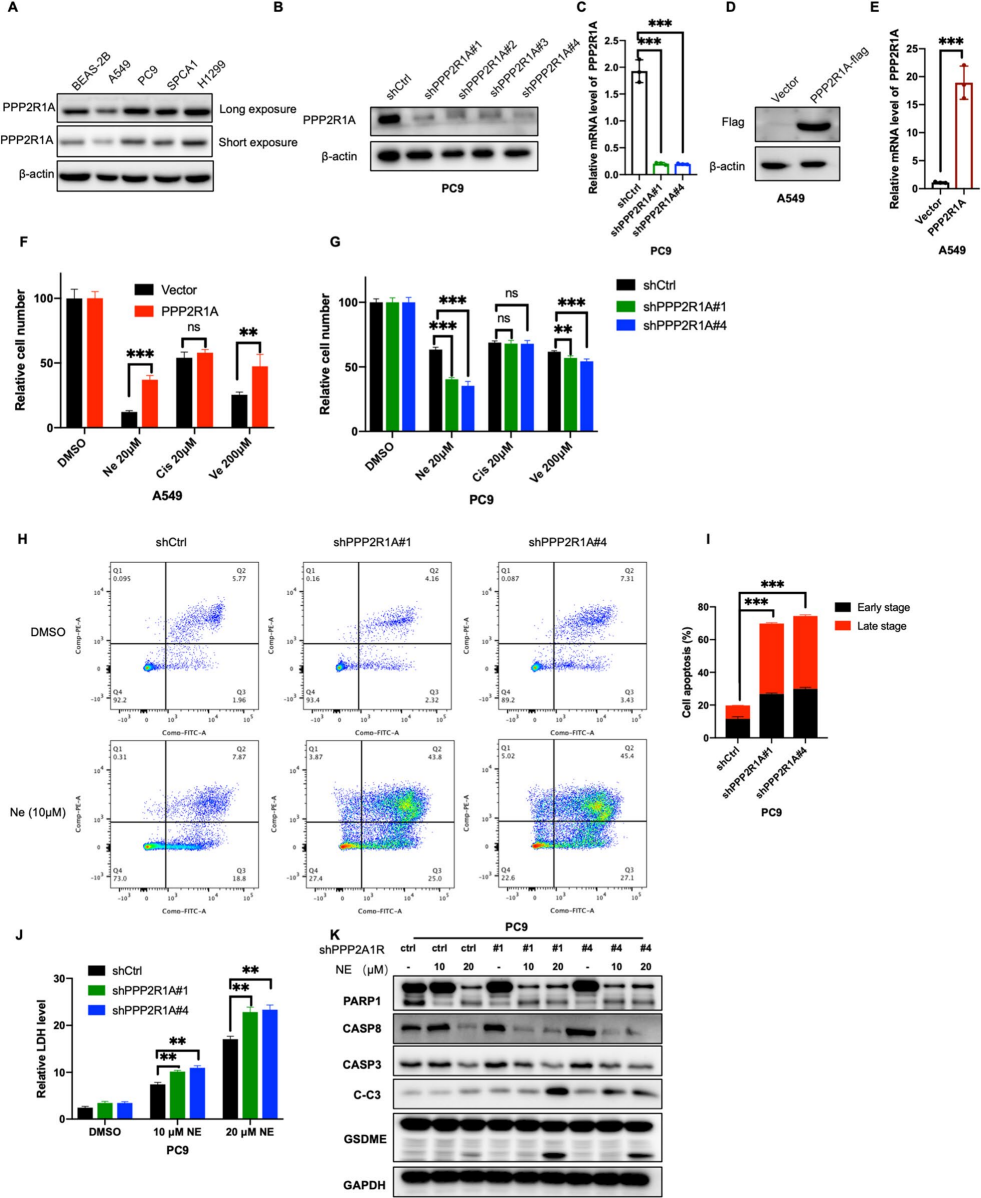
Knockdown PPP2R1A sensitizes cancer cells to nelfinavir treatment. **A** Expression of PPP2R1A in normal lung epithelial cells (BEAS-2B) and lung adenocarcinoma cells (A549, PC9, SPCA1 and H1299). **B**, **C** Protein (**B**) and mRNA (**C**) level of PPP2R1A were determined in PC9 knockdown PPP2R1A cell lines. **D**, **E** Protein (**D**) and mRNA (**E**) level of PPP2R1A were determined in A549 overexpressing PPP2R1A cell lines. **F** CCK8 assay measured the cell viability of A549 overexpressing PPP2R1A cell lines under the treatment of nelfinavir (20 μmol), cisplatin (20 μmol) or velnacrine (200 μmol) for 24 h. **G** CCK8 assay measured the cell viability of PC9 knockdown PPP2R1A cell lines under the treatment of nelfinavir (20 μmol), cisplatin (20 μmol) or velnacrine (200 μmol) for 24 h. **H** PC9 knockdown PPP2R1A cell lines under the treatment of nelfinavir (10 μmol) for 24 h. Apoptosis was detected by Annexin V-FITC/PI staining. **I** Percentages of early-stage and late-stage apoptotic cells among the three groups. **J** LDH release of PC9 knockdown PPP2R1A cell lines under the treatment of nelfinavir (10 μmol, 20 μmol) for 24 h. **K** Western blot detected apoptosis and pyroptosis-related protein in knockdown PPP2R1A cell lines

To further examine whether PPP2R1A drives tumor formation and drug resistance, we performed PPP2R1A-overexpression and PPP2R1A-knockdown experiments using lentiviral-based overexpression and knockdown approaches. Western blot analysis and RT-qPCR showed successful establishment of knockdown clones derived from PC9 cells which show a higher expression of PPP2R1A (Fig. 6A–C) and overexpression clones derived from A549 cells which show a lower expression of PPP2R1A (Fig. 6D, E). Interestingly, the overexpression of PPP2R1A decreased the effects of nelfinavir and velnacrine-induced cell death (Fig. 6F). In addition, knockdown of PPP2R1A sensitizes PC9 cells to nelfinavir and velnacrine treatment (Fig. 6G). Consistently, flow cytometry revealed that knockdown of PPP2R1A enhanced cell sensitivity to the treatment of nelfinavir and attenuated cell apoptosis (Fig. 6H, I). Nelfinavir treatment resulted in the cleavage of GSDME and Caspase3 (Fig. 6K). Next, LDH release was detected in knockdown PPP2R1A cell lines treated with/without nelfinavir. Significantly, knockdown of PPP2R1A promotes nelfinavir -induced LDH release (Fig. 6J). Moreover, the inhibition of PPP2R1A was found to enhance the cleavage of GSDME and Caspase3 in response to nelfinavir treatment (Fig. 6K). These results suggested that knockdown of PPP2R1A sensitizes cancer cells to nelfinavir treatment and contributes to nelfinavir induced cell apoptosis and pyroptosis.

## Discussion

Aberrant regulation of meiotic genes, closely associated with meiotic recombination, contributes to genome instability and ultimately leads to carcinogenesis [20]. Accompanied by aberrant alteration of meiotic genes, meiotic recombination exerts a profound influence on gene expression, tumor evolution [4, 21, 22] and therapy tolerance [23, 24]. Hence, we constructed a comprehensive and effective meiosis related prognostic model based on LASSO Cox regression analysis for the OS-related key meiotic genes to provide clinical prognostic information and diagnosis and treatment guidelines for LUAD patients.

In this study, we initially identified and analyzed meiotic related DEGs in TCGA database, and subsequently constructed a meiosis related prognostic model. Based on these findings, LUAD patients were divided into high-risk and low-risk subgroups. Notably, patients with higher had shorter survival time both in TCGA and GSE31210 database (Fig. 6D). Furthermore, we analyzed the relationship among the risk score, gender, smoking history, pathologic stage and the expression of the seven key meiotic DEGs. Consistently, PPP2R1A revealed higher expression in the high-risk group both in TCGA and GSE31210 database.

PPP2R1A emerged as the predominant factor in this model, suggesting its potential important role in LUAD progression. PPP2R1A, a well-characterized scaffold subunit of the PP2A complex [25], has been reported to decrease the cytotoxicity of chemoradiation treatment in pancreatic cancer via activating HRR and inhibiting CDC25/and CDK1 [26]. Additionally, PPP2R1A was identified as a migration regulator recently depends on its interaction with NHSL1-containing WAVE Shell Complex [25]. PPP2R1A also exerts crucial functions in mammalian brain development and function. Its dysfunction is one of the main reasons leads to neurodevelopmental disorder [27]. These observations underscore the multifaceted involvement of PPP2R1A across various cancers and neurodegenerative disease. In light of these insights, we initially focused on PPP2R1A, and found that PPP2R1A was significantly upregulated in LUAD tumor tissues through both TCGA database and clinical samples. Importantly, patients exhibiting high levels of PPP2R1A expression displayed shorter overall survival compared to those with low-expression counterparts.

cMAP analysis is a valuable resource for identifying relationships between diseases, genes, and chemicals. The correlation between genes and 2837 chemicals in 9 tumor cell lines was determined using cMAP. Notably, Nelfinavir and Velnacrine exhibited significant effects on the risk model and PPP2R1A, suggesting their potential contribution to lung cancer treatment. Nelfinavir, an orally administered protease inhibitor [27], has been found to show anti-cancer effects through various paths such as cell cycle regulation, apoptosis, autophagy, oxidative stress and the tumor microenvironment [27–30]. Clinical trials have also demonstrated that nelfinavir combined with radiation therapy is well tolerated and exerts clinical improvements in non-small cell lung cancer, pancreatic cancer and advanced rectal cancer [30–32]. Our study further elucidated that nelfinavir causes caspase-dependent apoptosis and GSDME-dependent pyroptosis. Moreover, combination treatment of nelfinavir with cisplatin synergistically inhibits growth and enhances cell death in lung cancer cells. Additionally, our results indicate that knockdown of PPP2R1A enhances nelfinavir-induced cell death while overexpression of PPP2R1A confers protection against chemical damage. Collectively, our findings provide novel insights into the mechanism underlying nelfinavir-induced cancer cell death and validate PPP2R1A as a promising therapeutic target for LUAD.

## Materials and methods

### Cell culture, plasmids and shRNAs

Human lung cancer cell line A549 (ATCC: CCL-185™) was obtained from ATCC. PC9 cell line was kindly provided by Professor S.W. Tsao, University of Hong Kong, Pokfulam, Hong Kong. 293T cells were maintained in DMEM (Gibco); other cells were maintained in RPMI 1640 (Gibco). All media was supplemented with 10% (V/V) FBS. All cell lines were cultured under 37 °C with 5% CO_2_.

PPP2R1A overexpressing plasmid was obtained from Geneppl. Lentiviral shRNA clones targeting Human PPP2R1A (#1 ACCAGGATGTGGACGTCAAAT; #2 CTCATAGACGAACTCCGCAAT; #3 TTGCCAATGTCCGCTTCAATG; #4 GTTCTTTGATGAGAAACTTAA) and the nontargeting control construct were purchased from Clontech (NO. 632177). Lentiviral particles were produced in 293T cells. A549 and PC9 cell lines that were transfected and selected with puromycin at a concentration of 2 μg/ml.

### CCK8 assay and flow cytometry

Exponentially growing cell lines were digested and seeded into 96-well plates with 1 × 10^4^ cells/well. After treatment with different concentrations of nelfinavir (Targetmol, T7779), velnacrine (Targetmol, T35045) and cisplatin (Targetmol, T1564) in different times points, cell viability was detected by SuperKine™ Maximum Sensitivity Cell Counting Kit-8 (CCK-8) (Abbkine, Cat#BMU106-CN). The 50% inhibitory concentration (IC50) of drug was calculated with the method of “log(inhibitor) vs. normalized response- Variable slope” using GraphPad Prism 8.0

Apoptosis were detected with the Annexin V-FITC/PI Apoptosis Detection Kit (Yeasen, NO. 40302), performed according to the manufacturer’s instructions. Cells were analyzed by flow cytometry (Fortessa, BD Biosciences).

### Quantitative real-time PCR assay

We executed qRT-PCR assay according to the methods described previously [33]. In brief, we extracted total RNA from samples using RNAfast 200 Kit (Fastagen, China, No. 220010). cDNA was synthesized by RNA (1 μg) using a TransScript® All-in-One First-Strand cDNA Synthesis SuperMix for qPCR (One-Step gDNA Removal) Kit (Transgen, China, No. AT341). Amplification and semiquantification of transcripts were performed using the SYBR Green mix (Biomake) and specific primers on a 7500 Fast Real Time PCR System (Applied Biosystems, Life Technologies). The primers sequences were as follows: PPP2R1A (F: ACCGCATGACTACGCTCTTCTG, R: TTGAAGCGGACATTGGCAACCG).

### Western blot and antibody

Details of these procedures have been previously described [33, 34]. The following antibodies were used: PPP2R1A (Proteintech, 15882–1-AP, 1:1000), Flag (Proteintech, 66008-4-Ig, 1:1000), GAPDH (Sangon Biotech, D190090, 1:1000), MLKL (Cell signaling technology, 37333, 1:1000), GSDME (Abcam, ab21591, 1:1000), PARP1 (Sangon Biotech, D161071, 1:1000), Caspase 3 (Cell signaling technology, 9662, 1:1000), Caspase 8 (Cell signaling technology, 4927, 1:1000), cleaved-Caspase-3 (Cell signaling technology, 9661, 1:1000).

### Data acquisition and analysis

Data of LUAD samples from TCGA and GSE31210 were acquired for subsequent analyses after excluding patients with incomplete clinical information [35]. GO analysis and GSVA analysis were performed on DEGs by R package “cluster Profiler” in this study [35].

Multivariate cox regression was used to screen meiosis-related genes. These genes were identified by LASSO analysis and consensus clustering divided the cohort into two groups [35]. The risk score for each patient was calculated as follows: risk score = sum (each candidate gene expression × corresponding LASSO regression coefficient).

### Statistical analyses

Results are shown as the mean ± SEM or SD. Significant differences between two groups were analyzed by unpaired Student’s t-test (two-tailed) if data were normally distributed; otherwise, data were analyzed by Kolmogorov–Smirnov test. All statistical analyses were performed using Prism 8.0 GraphPad software. A p-value < 0.05 was considered statistically significant.

### Supplementary Information

**Additional file 1: Figure S1.** UMAP analysis of grouped samples in TCGA and GSE31210. **Figure S2.** Nelfinavir and velnacrine were discovered by cMap.

## Data Availability

All data generated or analyzed during this study are included in this published article.

## References

[1] Denisenko T, Budkevich I, Zhivotovsky B (2018). Cell death-based treatment of lung adenocarcinoma. Cell Death Dis.

[2] Tan A, Tan D (2022). Targeted therapies for lung cancer patients with oncogenic driver molecular alterations. J Clin Oncol.

[3] Seguin L, Durandy M, Feral C (2022). Lung adenocarcinoma tumor origin: a guide for personalized medicine. Cancers.

[4] Liu C, Moten A, Ma Z, Lin H (2022). The foundational framework of tumors: Gametogenesis, p53, and cancer. Semin Cancer Biol.

[5] Baudat F, Imai Y, de Massy B (2013). Meiotic recombination in mammals: localization and regulation. Nat Rev Genet.

[6] Szostak J, Orr-Weaver T, Rothstein R, Stahl F (1983). The double-strand-break repair model for recombination. Cell.

[7] Ui A, Chiba N, Yasui A (2020). Relationship among DNA double-strand break (DSB), DSB repair, and transcription prevents genome instability and cancer. Cancer Sci.

[8] Harding SM, Boiarsky JA, Greenberg RA (2015). ATM dependent silencing links nucleolar chromatin reorganization to DNA damage recognition. Cell Rep.

[9] Pankotai T, Bonhomme C, Chen D, Soutoglou E (2012). DNAPKcs-dependent arrest of RNA polymerase II transcription in the presence of DNA breaks. Nat Struct Mol Biol.

[10] Boukaba A, Liu J, Ward C, Wu Q, Arnaoutov A, Liang J, Pugacheva E, Dasso M, Lobanenkov V, Esteban M (2022). Ectopic expression of meiotic cohesin generates chromosome instability in cancer cell line. Proc Natl Acad Sci USA.

[11] Gantchev J, Martínez Villarreal A, Gunn S, Zetka M, Ødum N, Litvinov IV (2020). The ectopic expression of meiCT genes promotes meiomitosis and may facilitate carcinogenesis. Cell Cycle.

[12] Gao Y, Kardos J, Yang Y, Tamir TY, Mutter-Rottmayer E, Weissman B, Major MB, Kim WY, Vaziri C (2018). The cancer/testes (CT) antigen HORMAD1 promotes homologous recombinational DNA repair and radioresistance in lung adenocarcinoma cells. Sci Rep.

[13] Chen YT, Venditti CA, Theiler G, Stevenson BJ, Iseli C, Gure AO, Jongeneel CV, Old LJ, Simpson AJ (2005). Identification of CT46/HORMAD1, an immunogenic cancer/testis antigen encoding a putative meiosis-related protein. Cancer Immun.

[14] Aung PP, Oue N, Mitani Y, Nakayama H, Yoshida K, Noguchi T, Bosserhoff AK, Yasui W (2006). Systematic search for gastric cancer-specific genes based on SAGE data: melanoma inhibitory activity and matrix metalloproteinase-10 are novel prognostic factors in patients with gastric cancer. Oncogene.

[15] Houle AA, Gibling H, Lamaze FC, Edgington HA, Soave D, Fave MJ, Agbessi M, Bruat V, Stein LD, Awadalla P (2018). Aberrant PRDM9 expression impacts the pan-cancer genomic landscape. Genome Res.

[16] Hussin J, Sinnett D, Casals F, Idaghdour Y, Bruat V, Saillour V, Healy J, Grenier JC, de Malliard T, Busche S (2013). Rare allelic forms of PRDM9 associated with childhood leukemogenesis. Genome Res.

[17] Ianzini F, Kosmacek EA, Nelson ES, Napoli E, Erenpreisa J, Kalejs M, Mackey MA (2009). Activation of meiosis-specific genes is associated with depolyploidization of human tumor cells following radiation-induced mitotic catastrophe. Cancer Res.

[18] Rivera M, Wu Q, Hamerlik P, Hjelmeland AB, Bao S, Rich JN (2015). Acquisition of meiotic DNA repair regulators maintain genome stability in glioblastoma. Cell Death Dis.

[19] Scully R, Panday A, Elango R, Willis NA (2019). DNA double-strand break repair-pathway choice in somatic mammalian cells. Nat Rev Mol Cell Biol.

[20] Sou I, Hamer G, Tee W, Vader G, McClurg U (2023). Cancer and meiotic gene expression: two sides of the same coin?. Curr Top Dev Biol.

[21] Zhang J, Gurusaran M, Fujiwara Y, Zhang K, Echbarthi M, Vorontsov E, Guo R, Pendlebury D, Alam I, Livera G (2020). The BRCA2-MEILB2-BRME1 complex governs meiotic recombination and impairs the mitotic BRCA2-RAD51 function in cancer cells. Nat Commun.

[22] Diederichs S, Bäumer N, Schultz N, Hamra F, Schrader M, Sandstede M, Berdel W, Serve H, Müller-Tidow C (2005). Expression patterns of mitotic and meiotic cell cycle regulators in testicular cancer and development. Int J Cancer.

[23] Morgan C, Nayak A, Hosoya N, Smith G, Lambing C (2023). Meiotic chromosome organization and its role in recombination and cancer. Curr Top Dev Biol.

[24] Hou H, Kyriacou E, Thadani R, Klutstein M, Chapman J, Cooper J (2021). Centromeres are dismantled by foundational meiotic proteins Spo11 and Rec8. Nature.

[25] Wang Y, Chiappetta G, Guérois R, Liu Y, Romero S, Boesch DJ, Krause M, Dessalles CA, Babataheri A, Barakat AI (2023). PPP2R1A regulates migration persistence through the NHSL1-containing WAVE Shell Complex. Nat Commun.

[26] Wei D, Parsels LA, Karnak D, Davis MA, Parsels JD, Marsh AC, Zhao L, Maybaum J, Lawrence TS, Sun Y (2013). Inhibition of protein phosphatase 2A radiosensitizes pancreatic cancers by modulating CDC25C/CDK1 and homologous recombination repair. Clin Cancer Res.

[27] Solomon BJ, Mok T, Kim DW (2014). First-line crizotinib versus chemotherapy in ALK-positive lung cancer. N Engl J Med.

[28] Perry C, Frampton J, McCormack P, Siddiqui M, Cvetković R (2005). Nelfinavir: a review of its use in the management of HIV infection. Drugs.

[29] Subeha M, Telleria C (2020). The anti-cancer properties of the HIV protease inhibitor nelfinavir. Cancers.

[30] Gills J, Lopiccolo J, Dennis P (2008). Nelfinavir, a new anti-cancer drug with pleiotropic effects and many paths to autophagy. Autophagy.

[31] Subeha M, Goyeneche A, Bustamante P, Lisio M, Burnier J, Telleria C (2021). Nelfinavir induces cytotoxicity towards high-grade serous ovarian cancer cells, involving induction of the unfolded protein response, modulation of protein synthesis, DNA damage, lysosomal impairment, and potentiation of toxicity caused by proteasome inhibition. Cancers.

[32] Xiang T, Du L, Pham P, Zhu B, Jiang S (2015). Nelfinavir, an HIV protease inhibitor, induces apoptosis and cell cycle arrest in human cervical cancer cells via the ROS-dependent mitochondrial pathway. Cancer Lett.

[33] Hill E, Roberts C, Franklin J, Enescu M, West N, MacGregor T, Chu K, Boyle L, Blesing C, Wang L (2016). Clinical trial of oral nelfinavir before and during radiation therapy for advanced rectal cancer. Clin Cancer Res.

[34] Al-Assar O, Bittner M, Lunardi S, Stratford M, McKenna W, Brunner T (2016). The radiosensitizing effects of Nelfinavir on pancreatic cancer with and without pancreatic stellate cells. Radiother Oncol.

[35] Liu Y, Ouyang L, Mao C, Chen Y, Li T, Liu N, Wang Z, Lai W, Zhou Y, Cao Y (2022). PCDHB14 promotes ferroptosis and is a novel tumor suppressor in hepatocellular carcinoma. Oncogene.

[36] Liu Y, Mao C, Wang M, Liu N, Ouyang L, Liu S, Tang H, Cao Y, Liu S, Wang X (2020). Cancer progression is mediated by proline catabolism in non-small cell lung cancer. Oncogene.

[37] Liang L, He H, Jiang S, Liu Y, Huang J, Sun X, Li Y, Jiang Y, Cong L (2022). TIAM2 contributes to osimertinib resistance, cell motility, and tumor-associated macrophage m2-like polarization in lung adenocarcinoma. Int J Mol Sci.

