# Supplementary material for: PPP2R1A silencing suppresses LUAD progression by sensitizing cells to nelfinavir-induced apoptosis and pyroptosis
Source: Cancer Cell Int. 2024 Apr 23;24:145. doi: 10.1186/s12935-024-03321-5 (PMC11040972; doi:10.1186/s12935-024-03321-5)
Supplement: Supplementary file 1 — Additional file 1: Figure S1. UMAP analysis of grouped samples in TCGA and GSE31210. Figure S2. Nelfinavir and velnacrine were discovered by cMap. [file 12935_2024_3321_MOESM1_ESM.docx]

**PPP2R1A silencing suppresses LUAD progression by sensitizing cells to nelfinavir - induced apoptosis and pyroptosis**

Yating Liu^1#^，Lianlian Ouyang^1,2,3#^， Shiyao Jiang^4,5^, Lu Liang^4,5^, Yuanbing Chen^6^ , Chao Mao^7^, Yiqun Jiang^4.5^* Li Cong^4.5^*

1. Department of Pharmacy, The Third Xiangya Hospital, Central South University, Changsha, 410013, Hunan, China

2. Department of Dermatology, Hunan Key Laboratory of Medical Epigenomics, Second Xiangya Hospital, Central South University, 410011, Changsha, China.

3. Research Unit of Key Technologies of Diagnosis and Treatment for Immune-related Skin Diseases, Chinese Academy of Medical Sciences, 410011, Changsha, China.

4. The Key Laboratory of Model Animal and Stem Cell Biology in Hunan Province, Hunan Normal University, Changsha, 410013, Hunan, People's Republic of China.

5. School of Medicine, Hunan Normal University, Changsha, 410013, Hunan, People's Republic of China. jiangyiqun@hunnu.edu.cn. [congli@hunnu.edu.cn](mailto:congli@hunnu.edu.cn).

6. Department of Neurosurgery, The Third Xiangya Hospital, Central South University, Changsha, 410013, Hunan, China.

7. Department of Experimental Radiation Oncology, The University of Texas MD Anderson Cancer Center, Houston, TX, USA


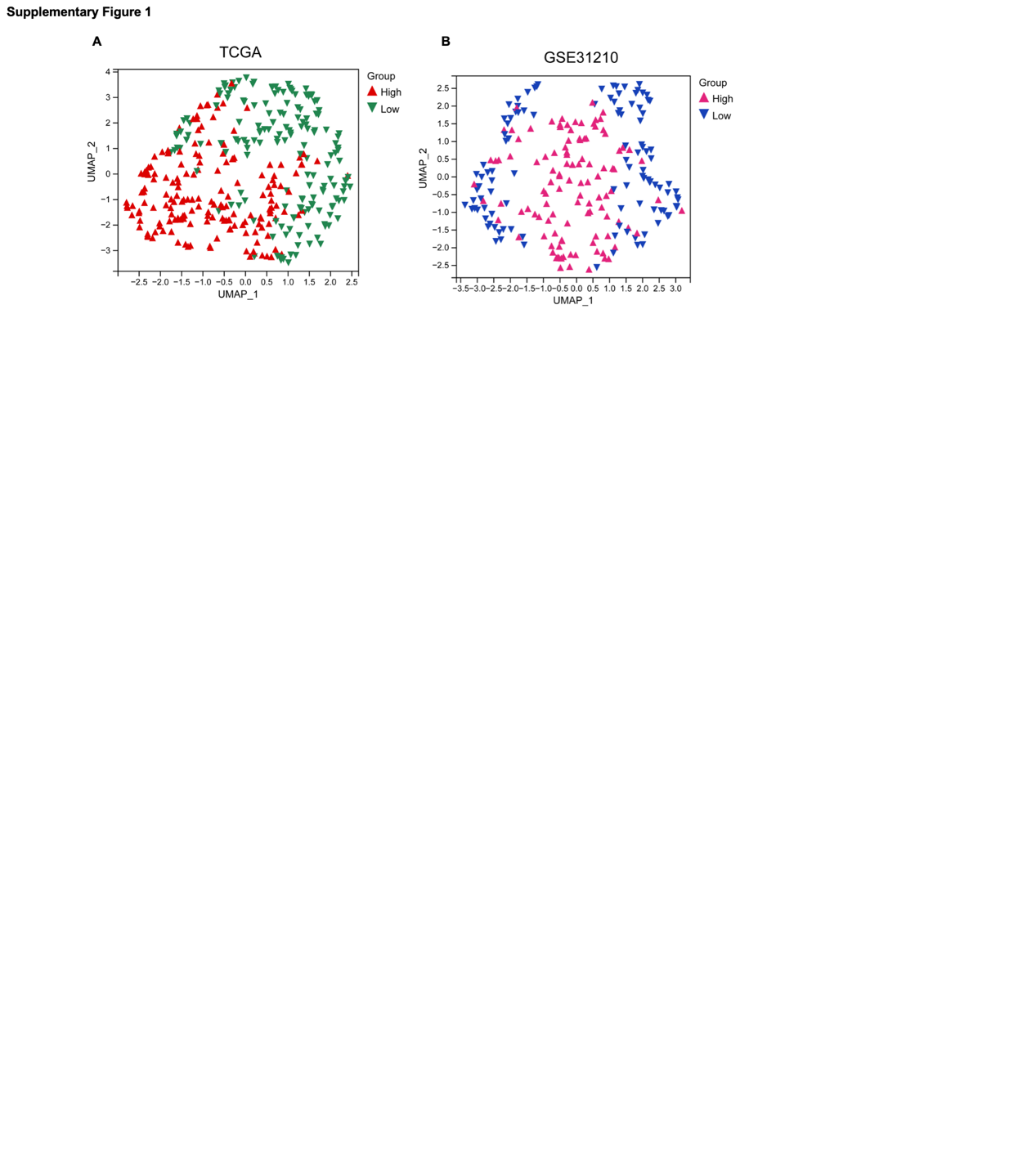


Figure S1. UMAP analysis of grouped samples in TCGA and GSE31210.


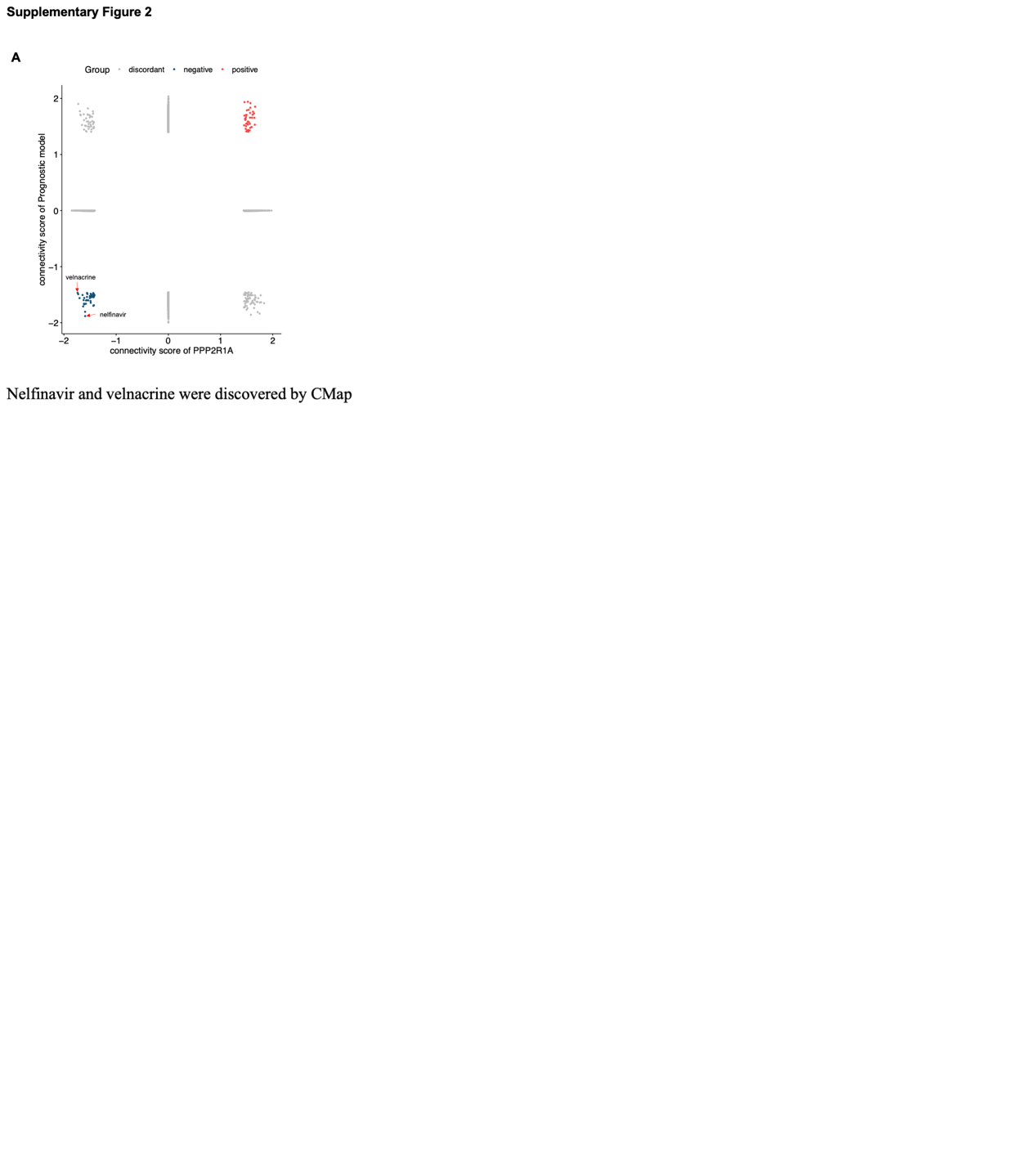


Figure S2. Nelfinavir and velnacrine were discovered by cMap.
